# Supplementary material for: Design and Synthesis In Silico Drug-like Prediction and Pharmacological Evaluation of Cyclopolymethylenic Homologous of LASSBio-1514
Source: Molecules. 2021 Aug 10;26(16):4828. doi: 10.3390/molecules26164828 (PMC8399892; doi:10.3390/molecules26164828)
Supplement: Supplementary file 1 [file molecules-26-04828-s001.zip › molecules-1299639-supplementary.pdf]

Suplementary Materials

Design and Synthesis in Silico Drug-Like Prediction and Pharmacological Evaluation of Cyclopolymethylenic Homologous of LASSBio-1514.

Lidia Moreira Lima [1,2,3,*], Tiago Fernandes da Silva [1,3], Carlos Eduardo da Silva Monteiro [2,4], Cristiane Aparecida-Silva [1,2], Walfrido Bispo Júnior [5], Aline Cavalcanti de Queiroz [5], Magna Suzana Alexandre-Moreira [5,], Gisele Zapata-Sudo [2,4] and Eliezer J. Barreiro [1,2,3*]

1 Instituto Nacional de Ciência e Tecnologia de Fármacos e Medicamentos (INCT-INOFAR; http://www.inct-inofar.ccs.ufrj.br/). Universidade Federal do Rio de Janeiro, Laboratório de Avaliação e Síntese de Substâncias Bioativas (LASSBio®, http://www.lassbio.icb.ufrj.br/) CCS, Cidade Universitária, P.O. Box 68024, Rio de Janeiro-RJ 21941-971, Brasil
2 Programa de Pós-graduação em Farmacologia e Química Medicinal, Instituto de Ciências Biomédicas, Universidade Federal do Rio de Janeiro, Rio de Janeiro, RJ 21941-902, Brasil
3 Programa de Pós-graduação em Química, Instituto de Química, Universidade Federal do Rio de Janeiro, Rio de Janeiro, RJ 21941-909, Brasil
4 Laboratório de Farmacologia Cardiovascular, Universidade Federal do Rio de Janeiro, Rio de Janeiro, RJ 21941-971, Brazil
5 LaFI—Laboratório de Farmacologia e Imunidade, Instituto de Ciências Biológicas e da Saúde, Universidade Federal de Alagoas, Maceió, AL 57072-900, Brasil

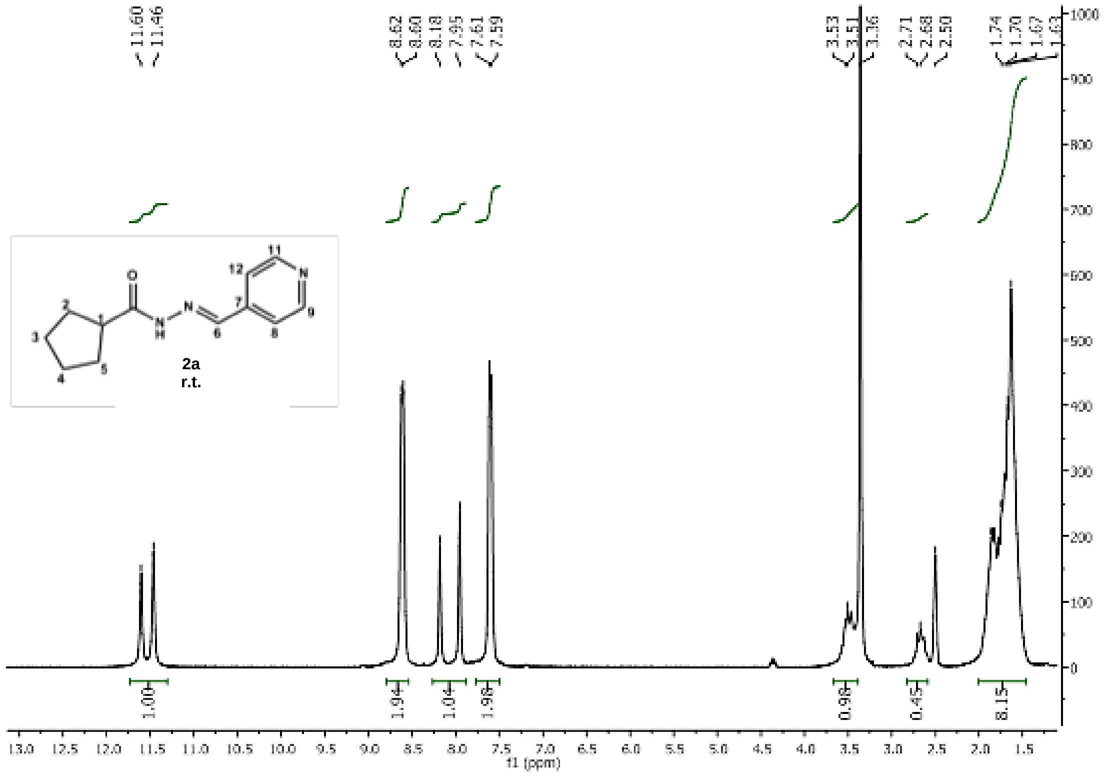

Figure S1. ^{1}H NMR spectrum of **2a** (DMSO-d$_6$, 200 MHz).

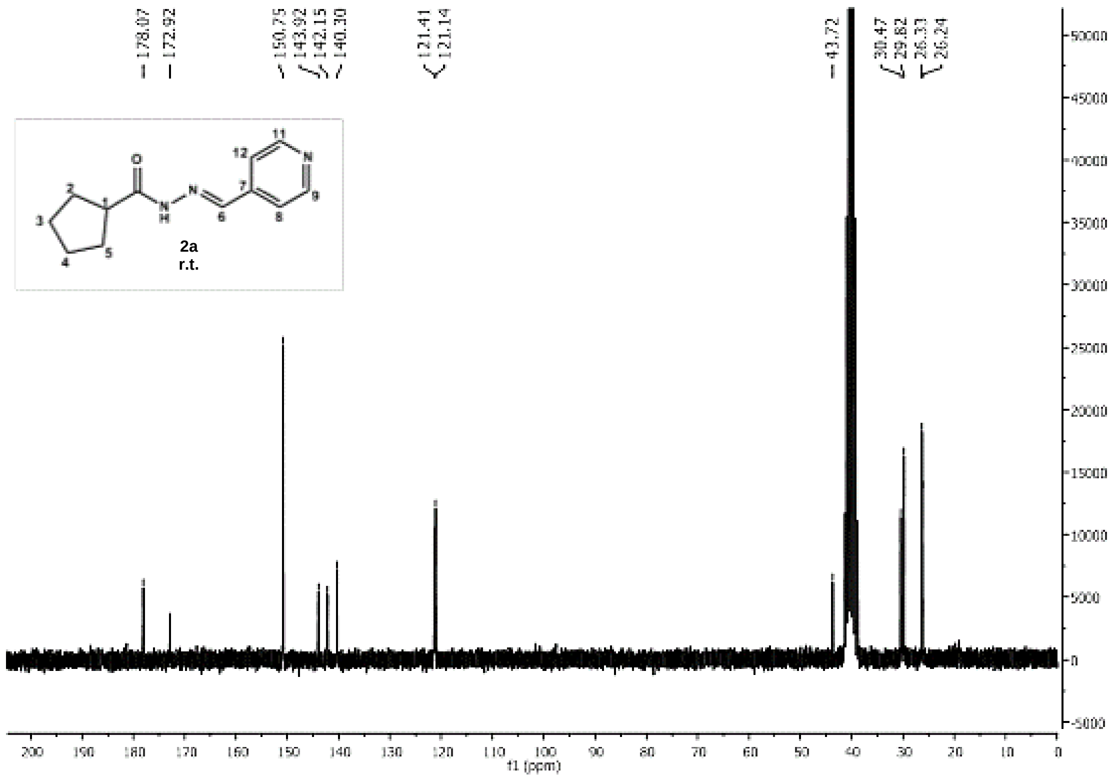

Figure S2. ^{13}C NMR spectrum of **2a** (DMSO-d_6, 50 MHz).

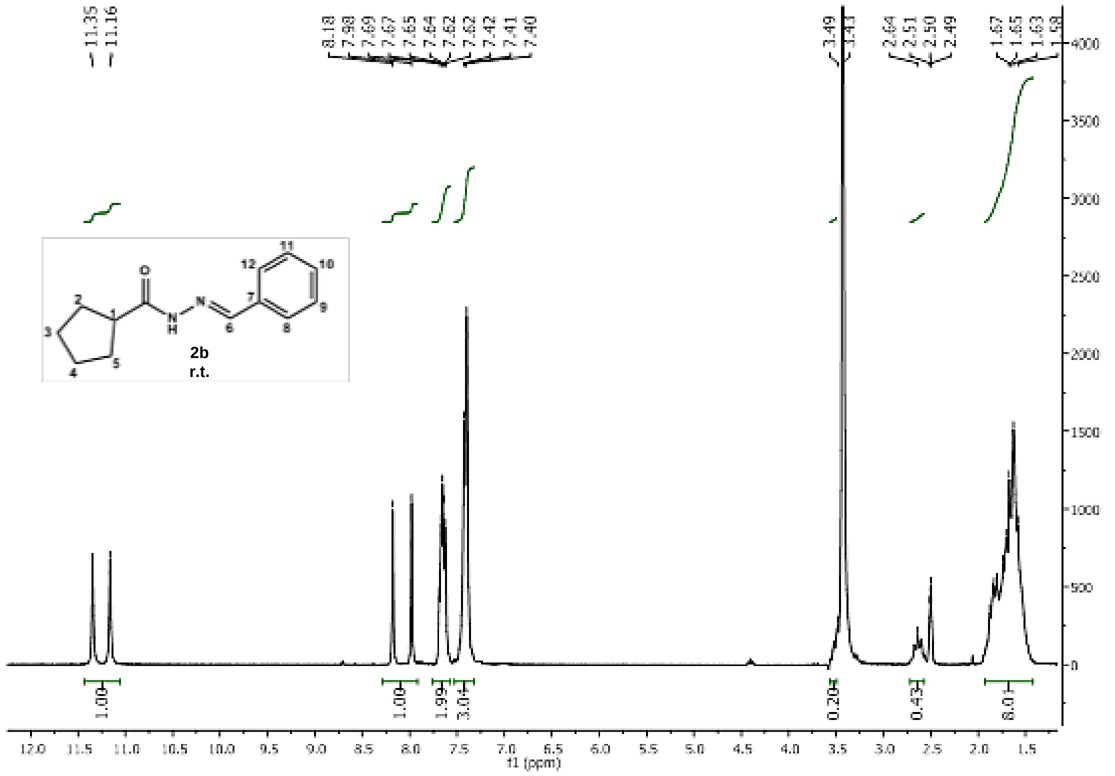

Figure S3. ^{1}H NMR spectrum of **2b** (DMSO-d_6, 200 MHz).

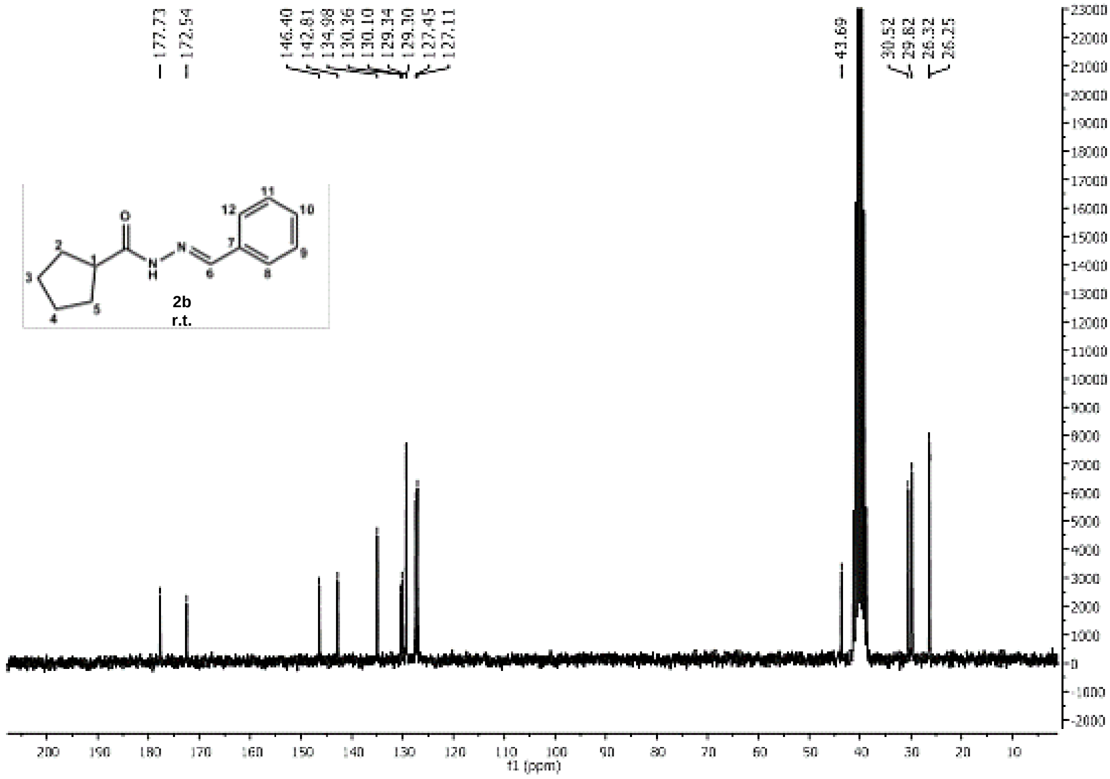

Figure S4. ^{13}C NMR spectrum of **2b** (DMSO-d_6, 50 MHz).

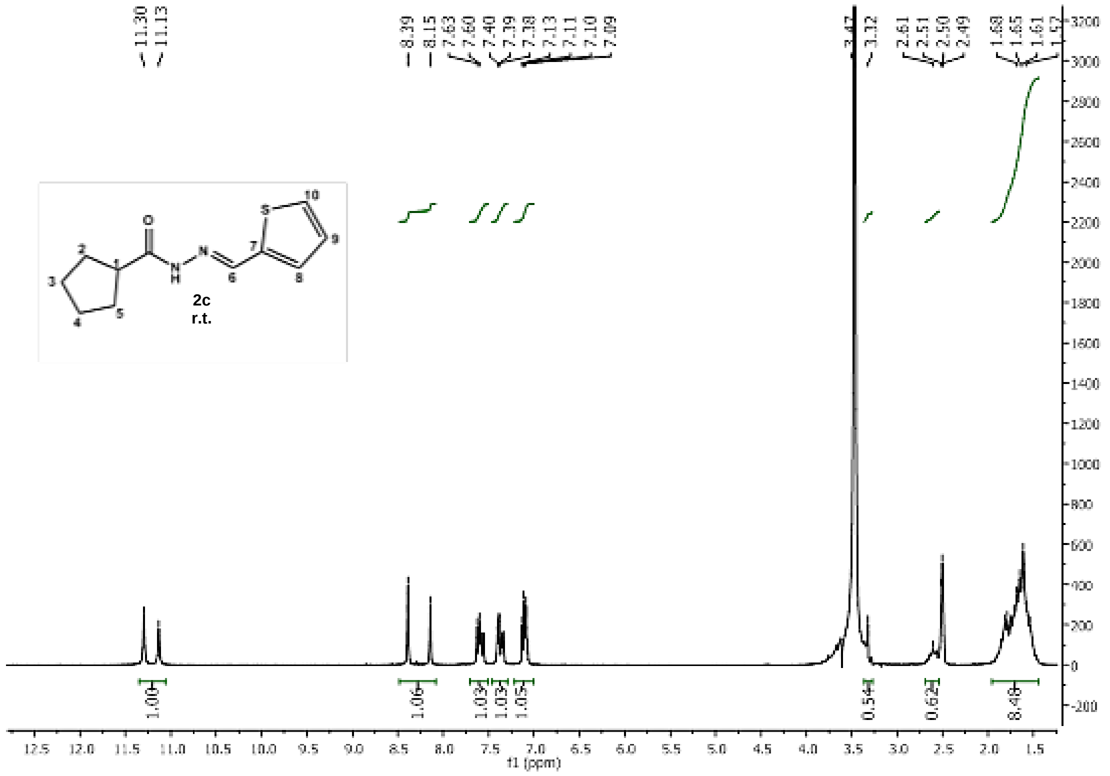

Figure S5. ^{1}H NMR spectrum of **2c** (DMSO-d_6, 200 MHz).

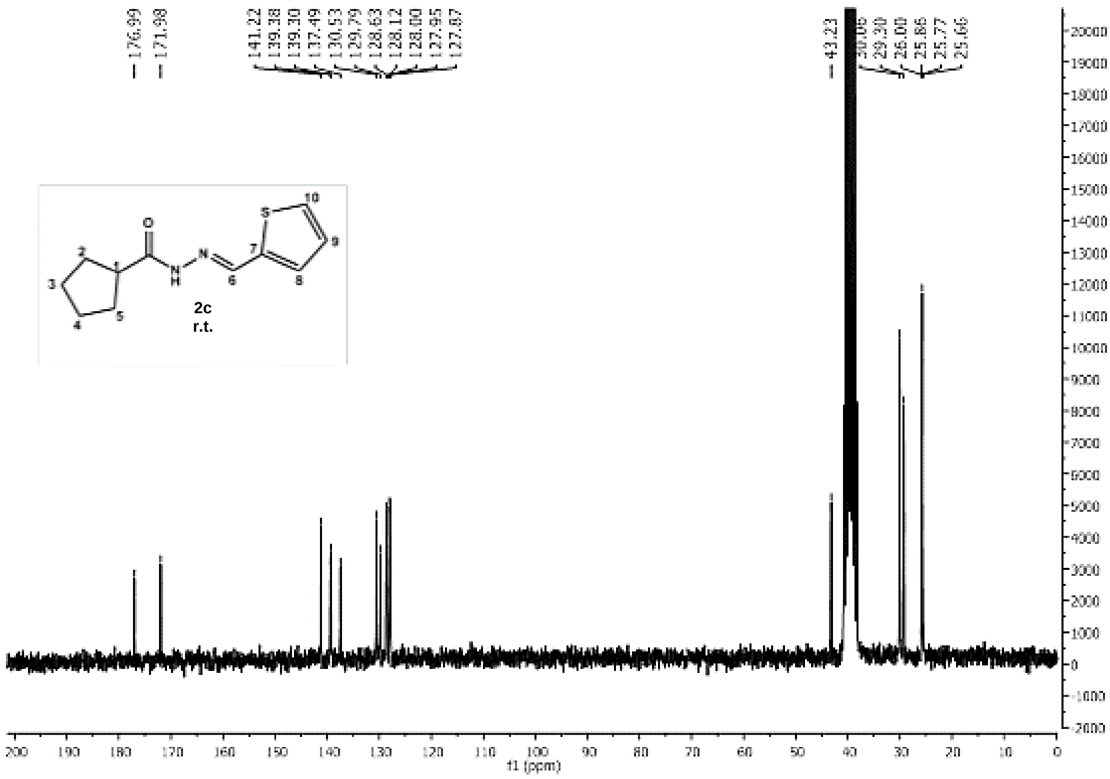

Figure S6. ^{13}C NMR spectrum of **2c** (DMSO-d_6, 50 MHz).

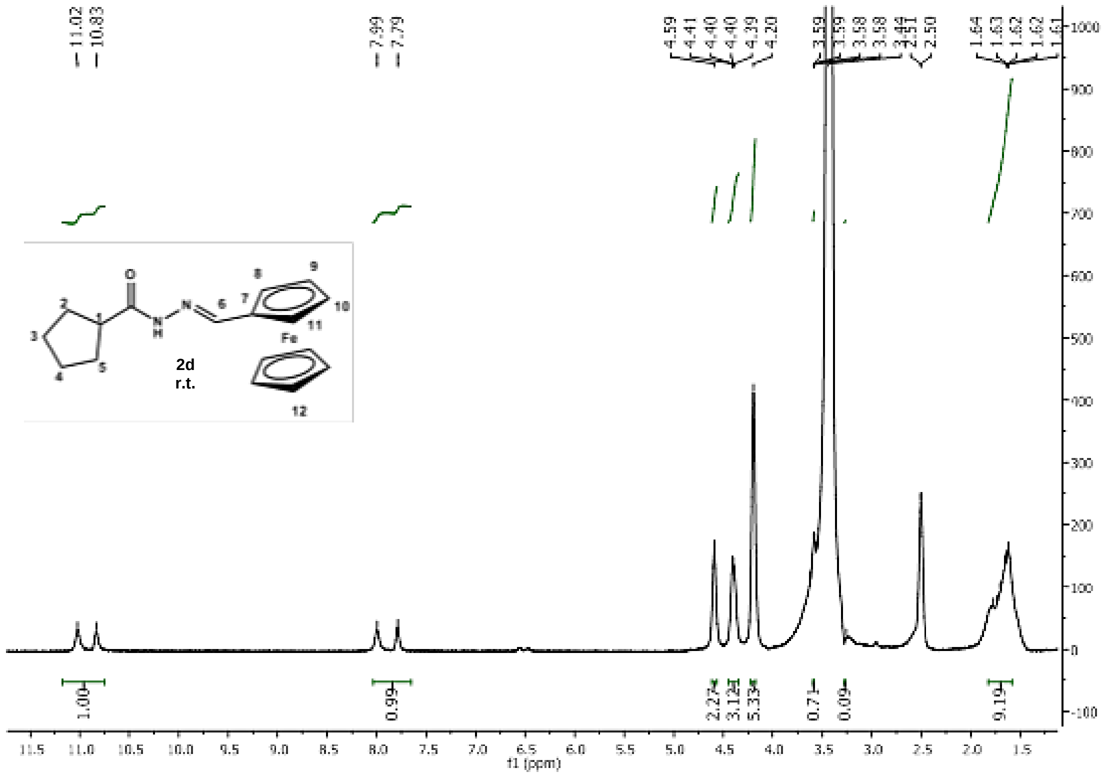

Figure S7. ^{1}H NMR spectrum of **2d** (DMSO-d_6, 200 MHz).

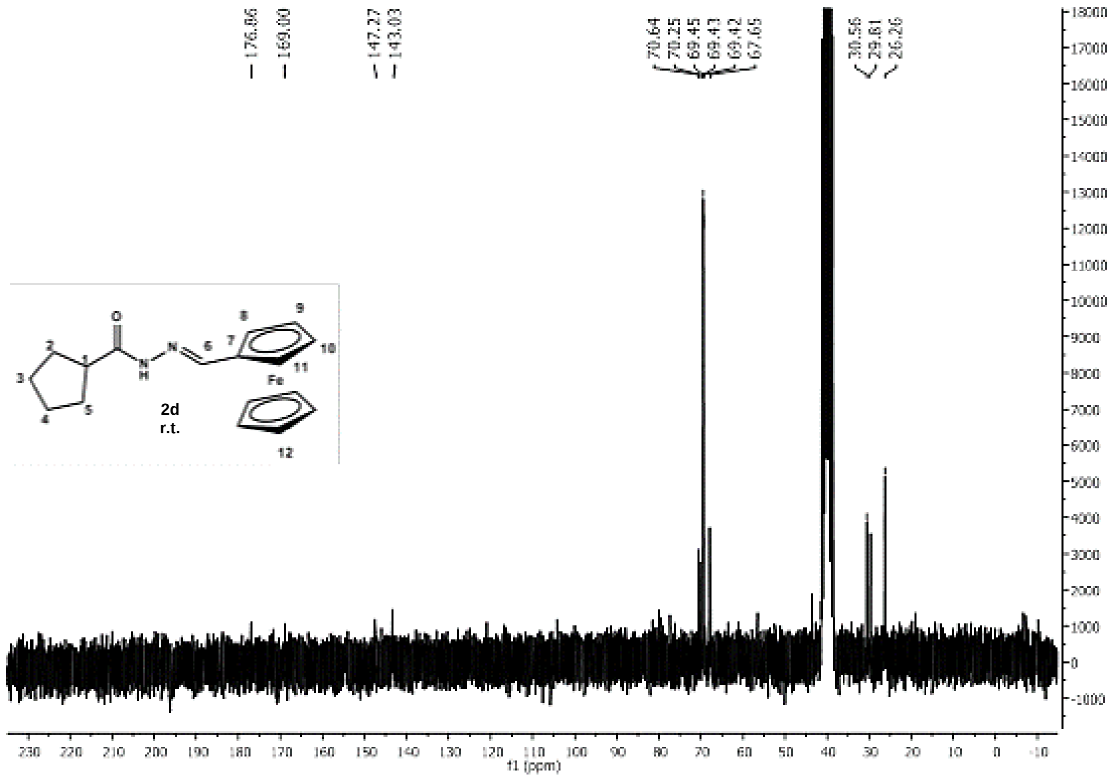

Figure S8. ^{13}C NMR spectrum of **2d** (DMSO-d_6, 50 MHz).

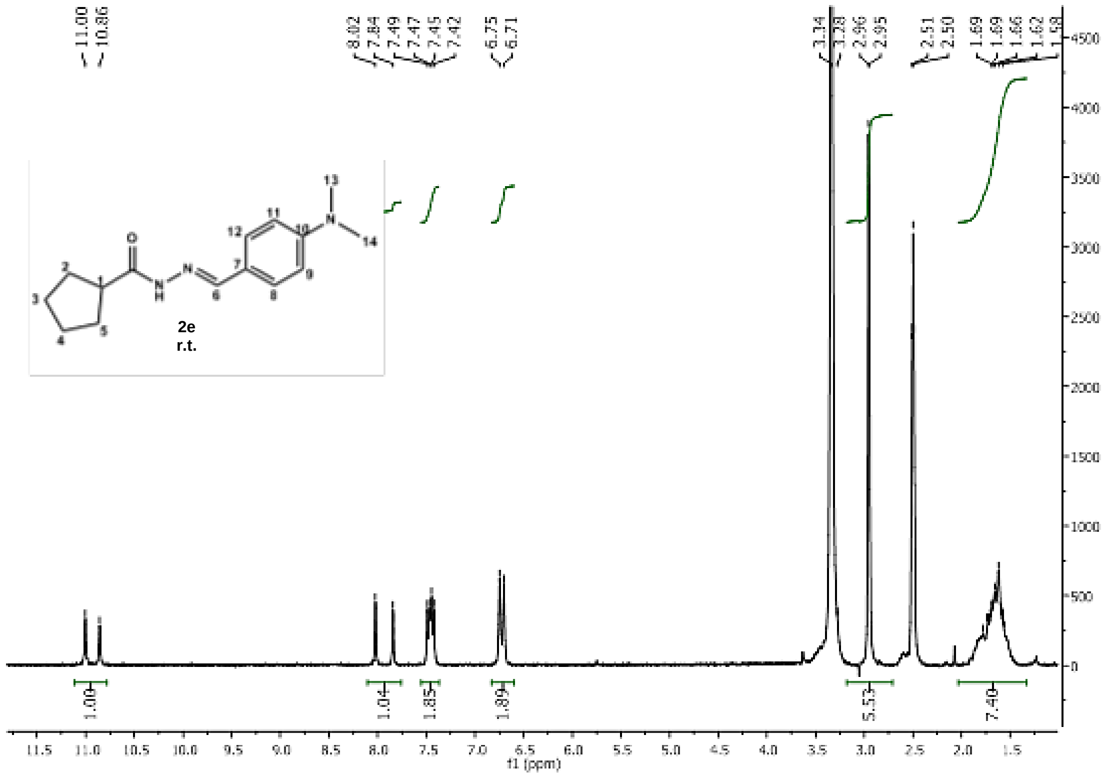

Figure S9. ^{1}H NMR spectrum of **2e** (DMSO-d_6, 200 MHz).

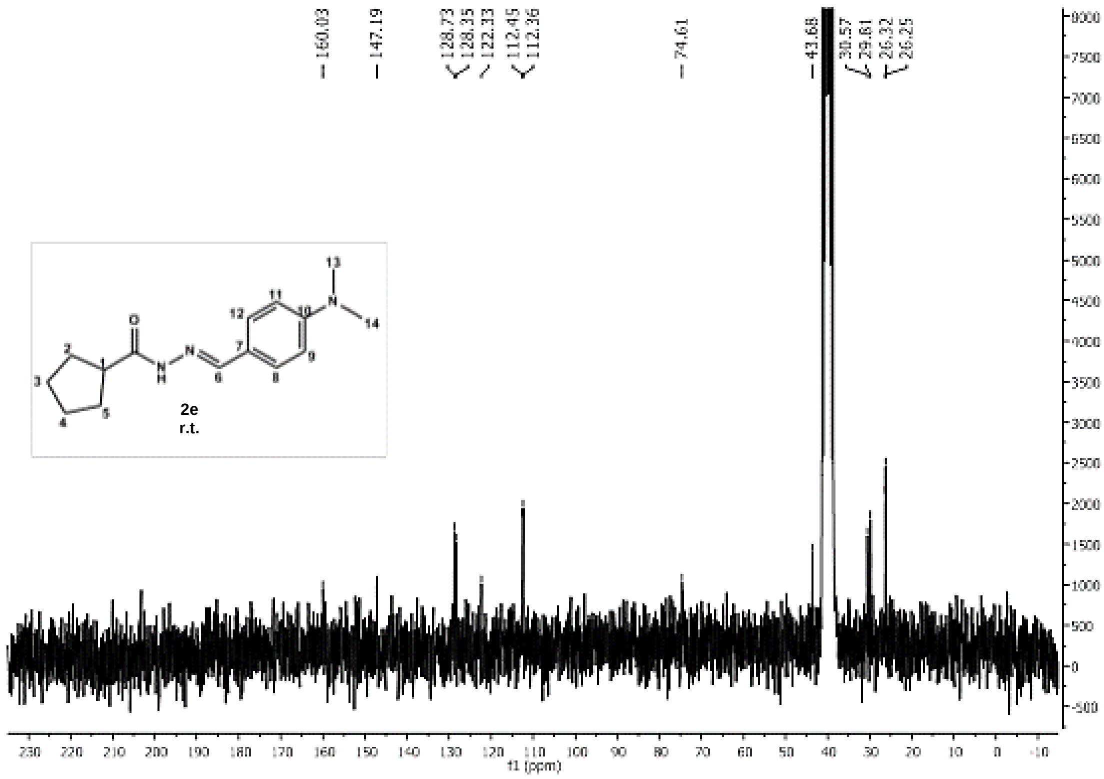

Figure S10. ^{13}C NMR spectrum of **2e** ($CDCl_3$, 50 MHz).

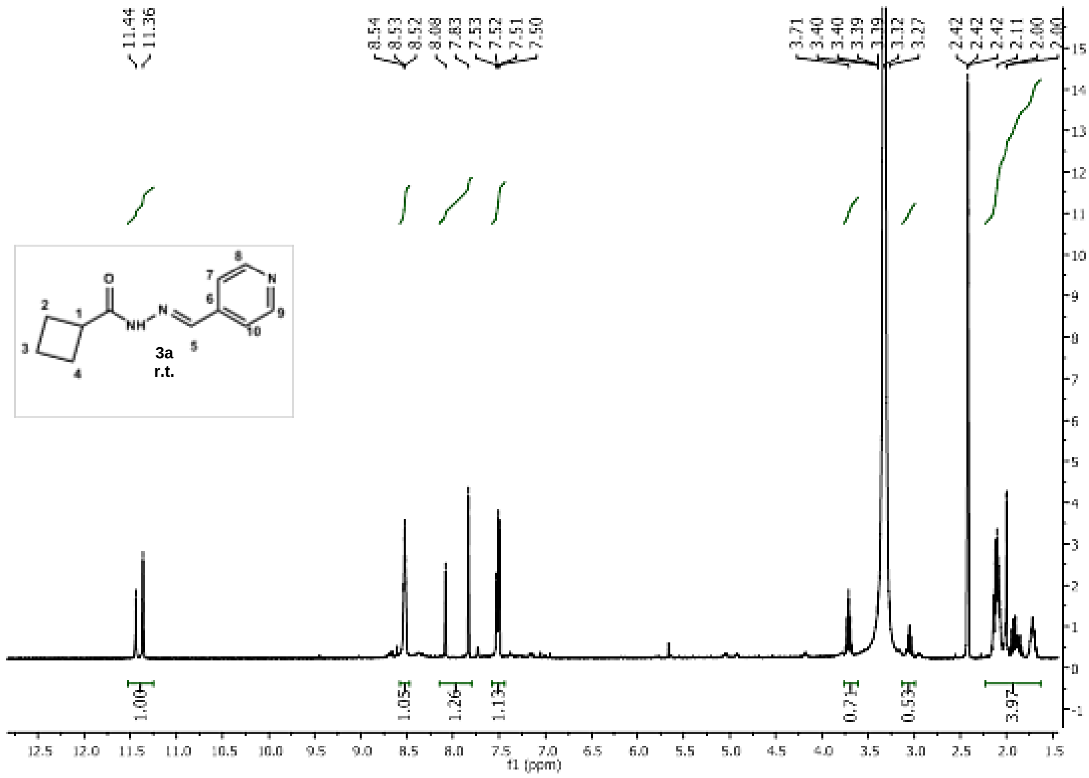

Figure S11. ^{1}H NMR spectrum of **3a** (DMSO-d_6, 200 MHz).

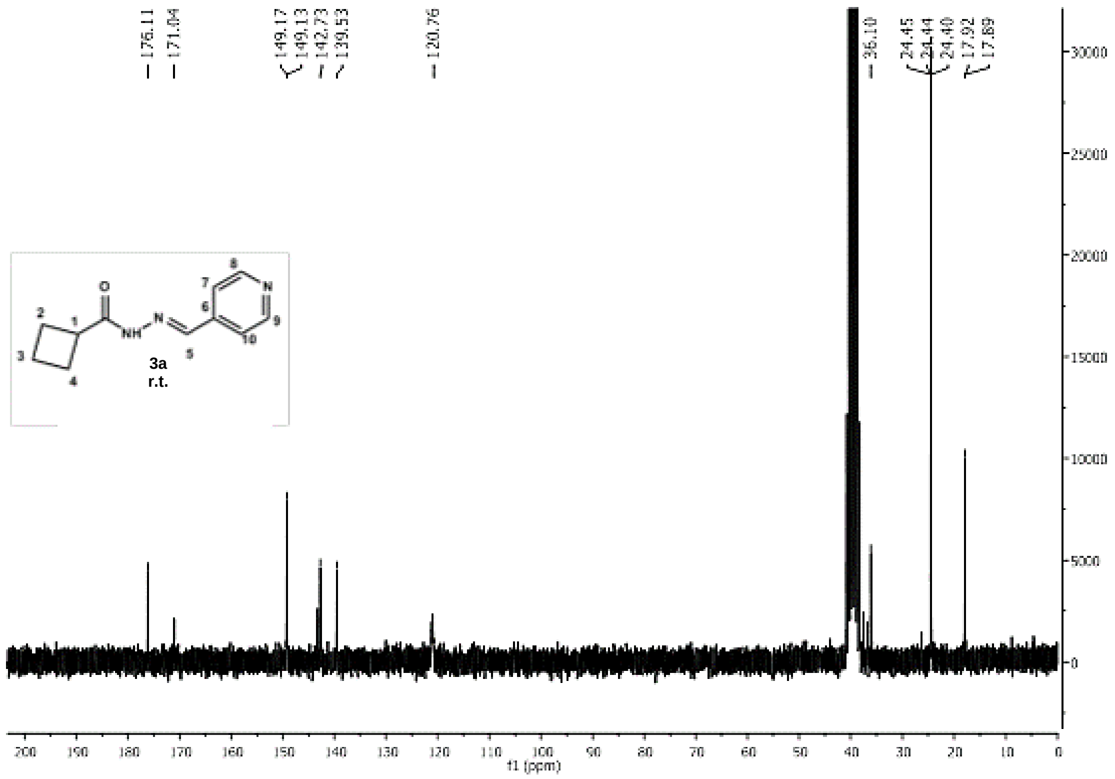

Figure S12. ^{13}C NMR spectrum of **3a** (DMSO-d_6, 50 MHz).

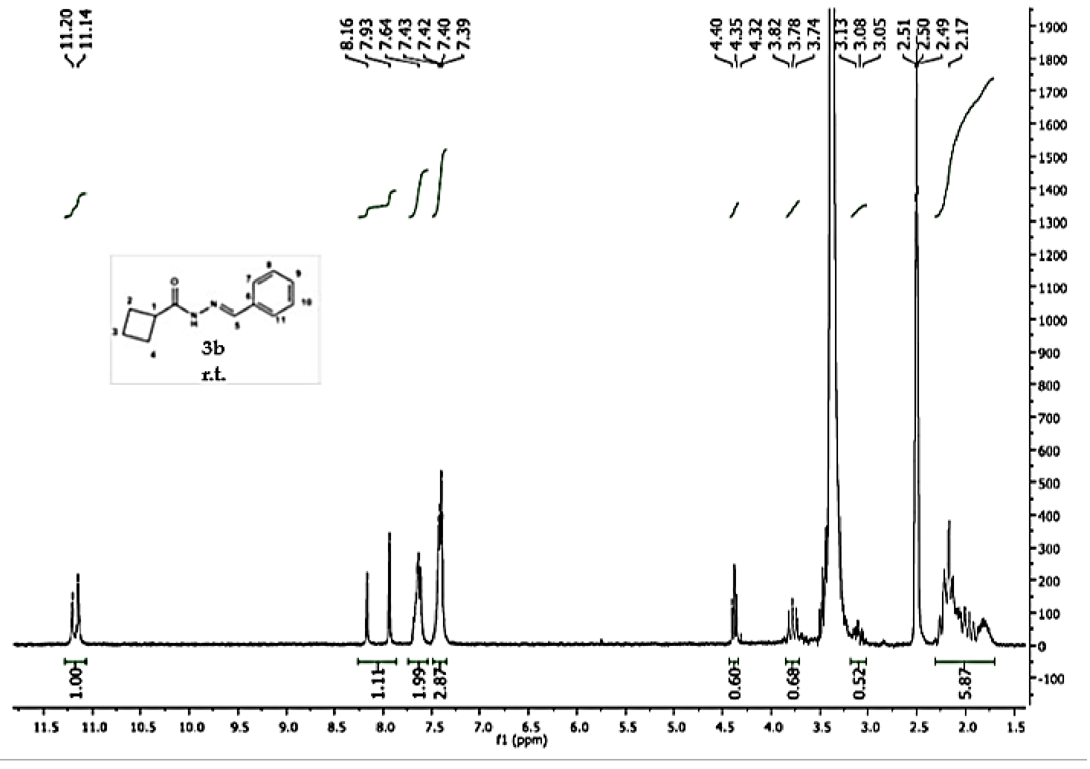

Figure S13. 1H NMR spectrum of 3b (DMSO-d6, 400 MHz).

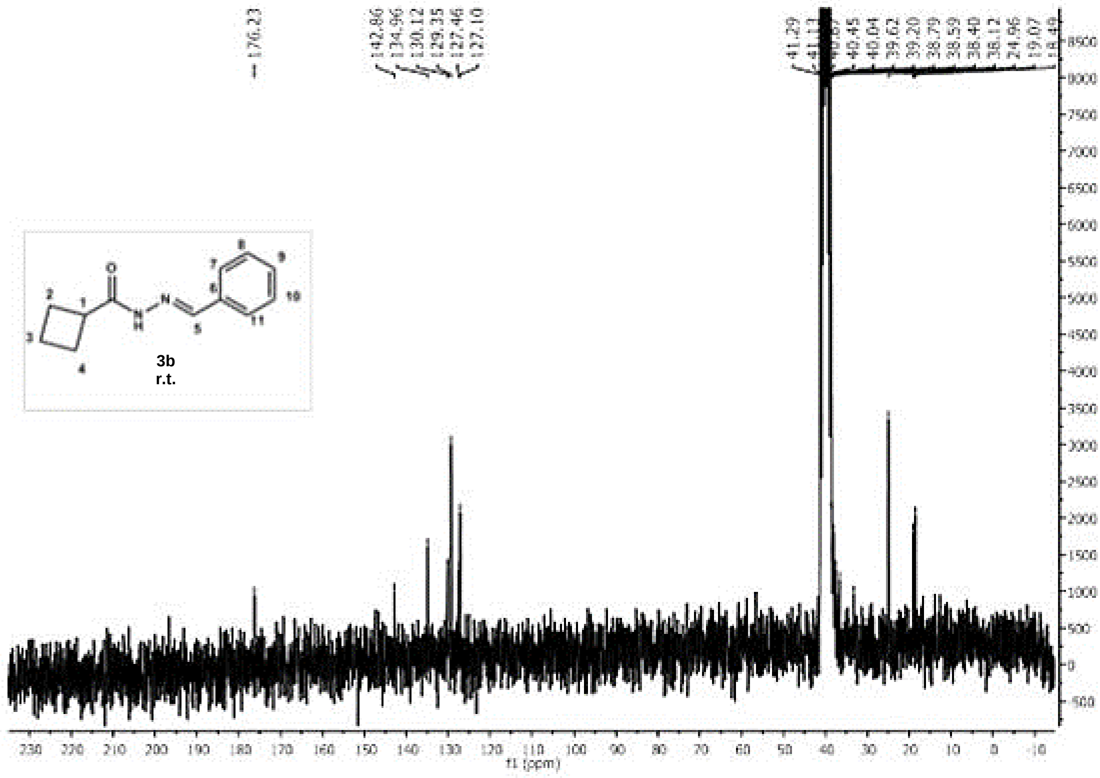

Figure S14. ^{13}C NMR spectrum of **3b** (DMSO-d$_6$, 50 MHz).

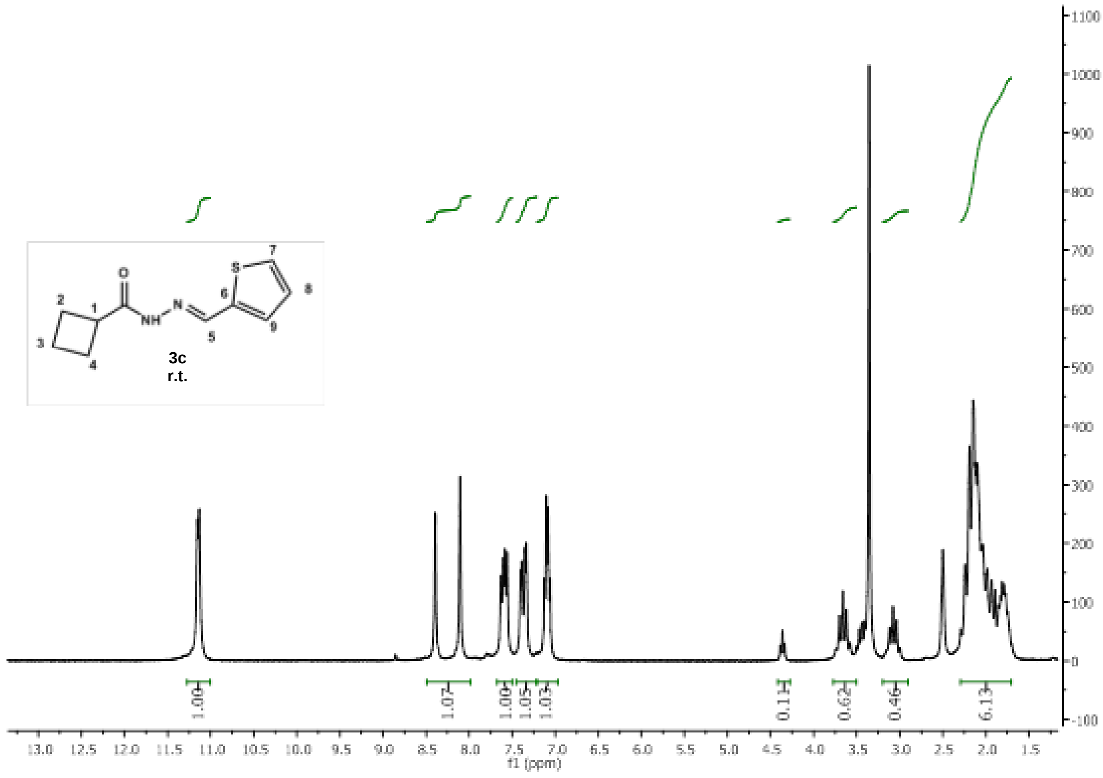

Figure S15. ^{1}H NMR spectrum of **3c** (DMSO-d$_6$, 200 MHz).

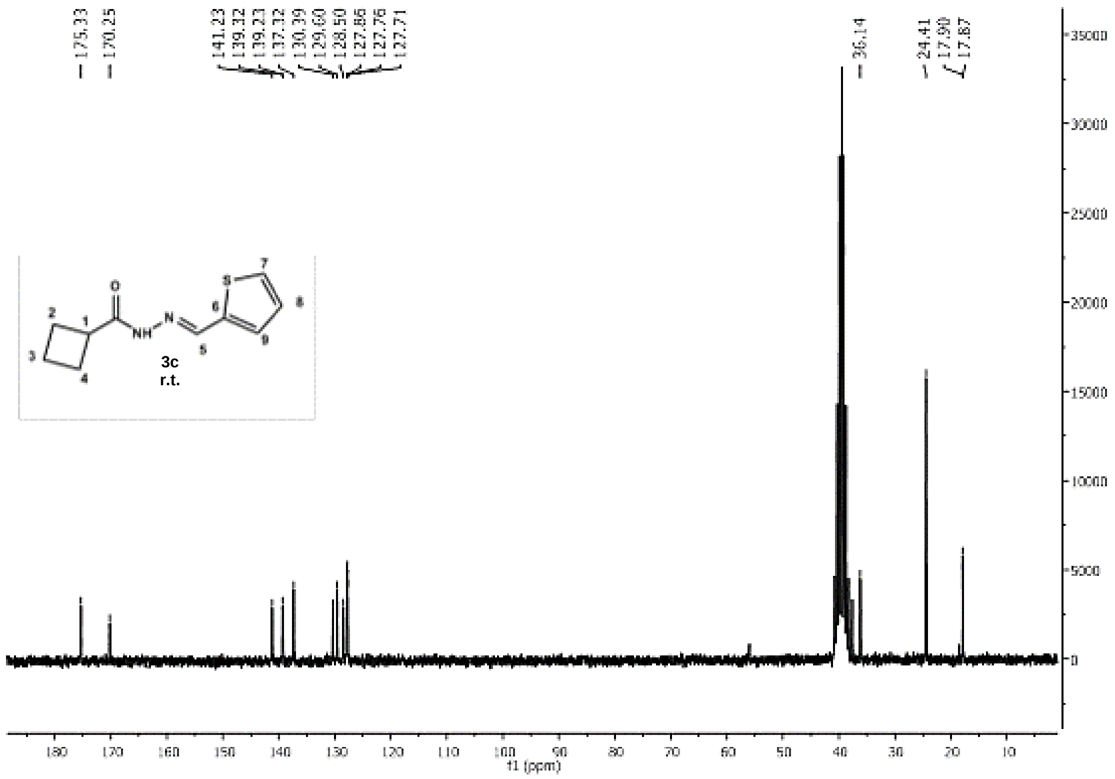

Figure S16. ^{13}C NMR spectrum of **3c** (DMSO-d$_6$, 50 MHz).

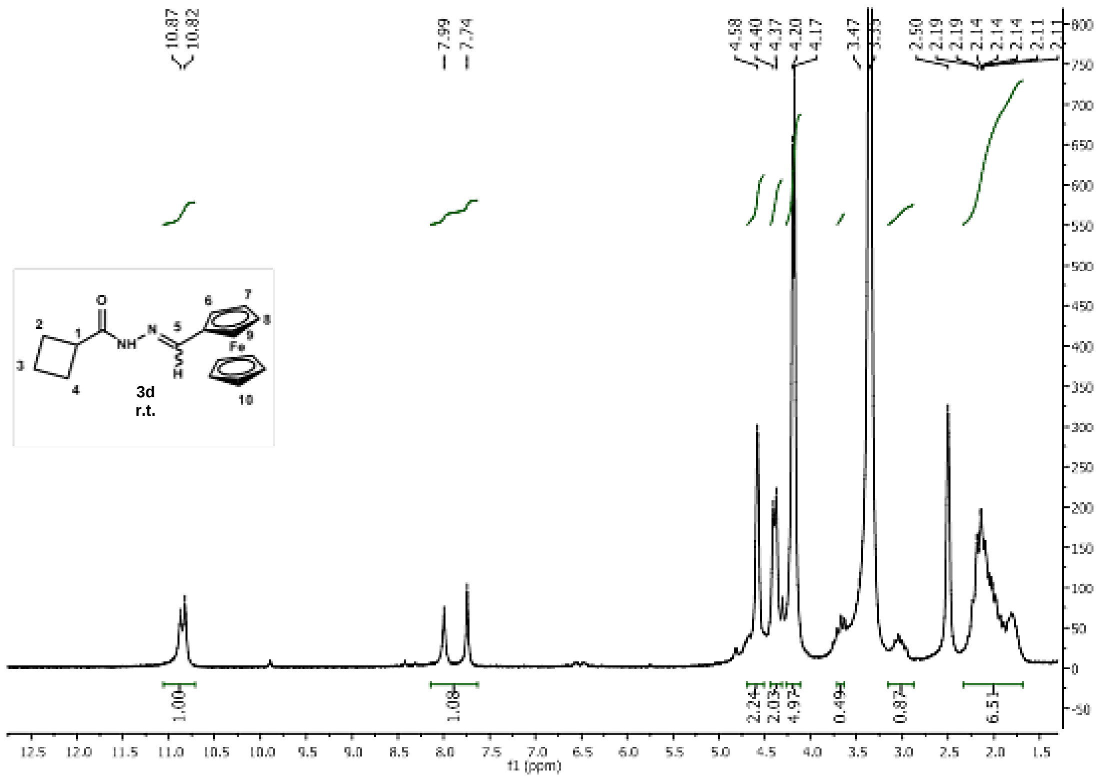

Figure S17. ^{1}H NMR spectrum of **3d** (DMSO-d$_6$, 200 MHz).

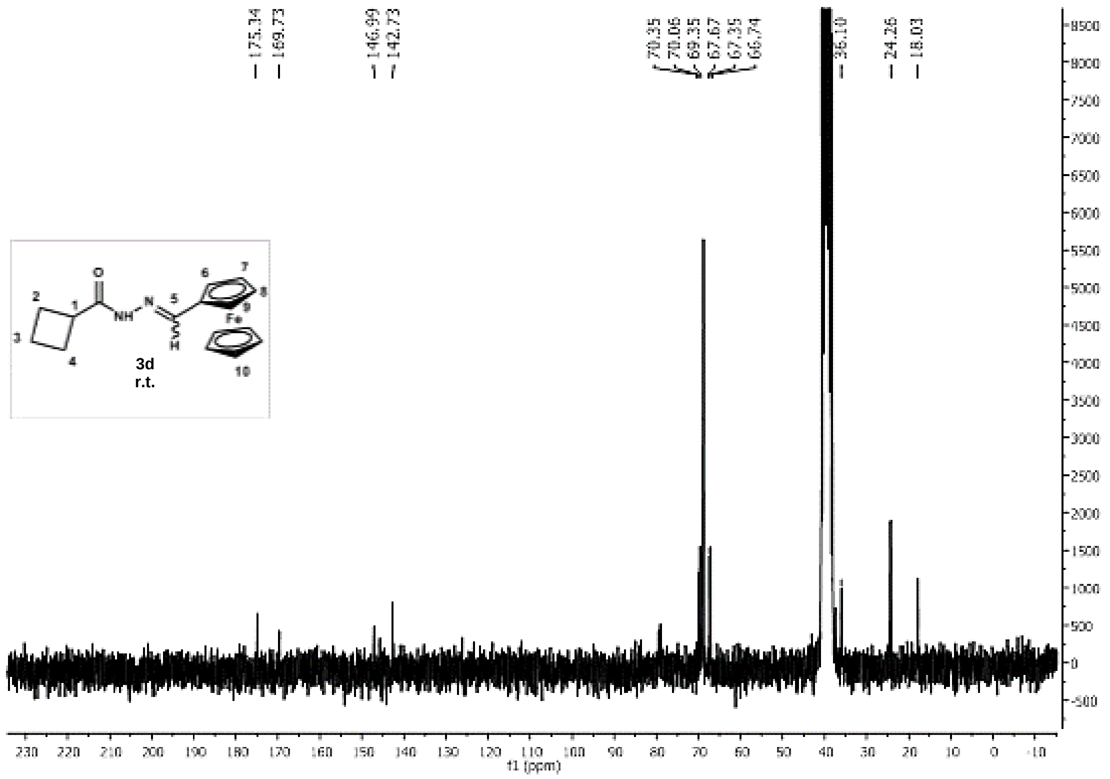

Figure S18. ^{13}C NMR spectrum of **3d** (DMSO-d$_6$, 50 MHz).

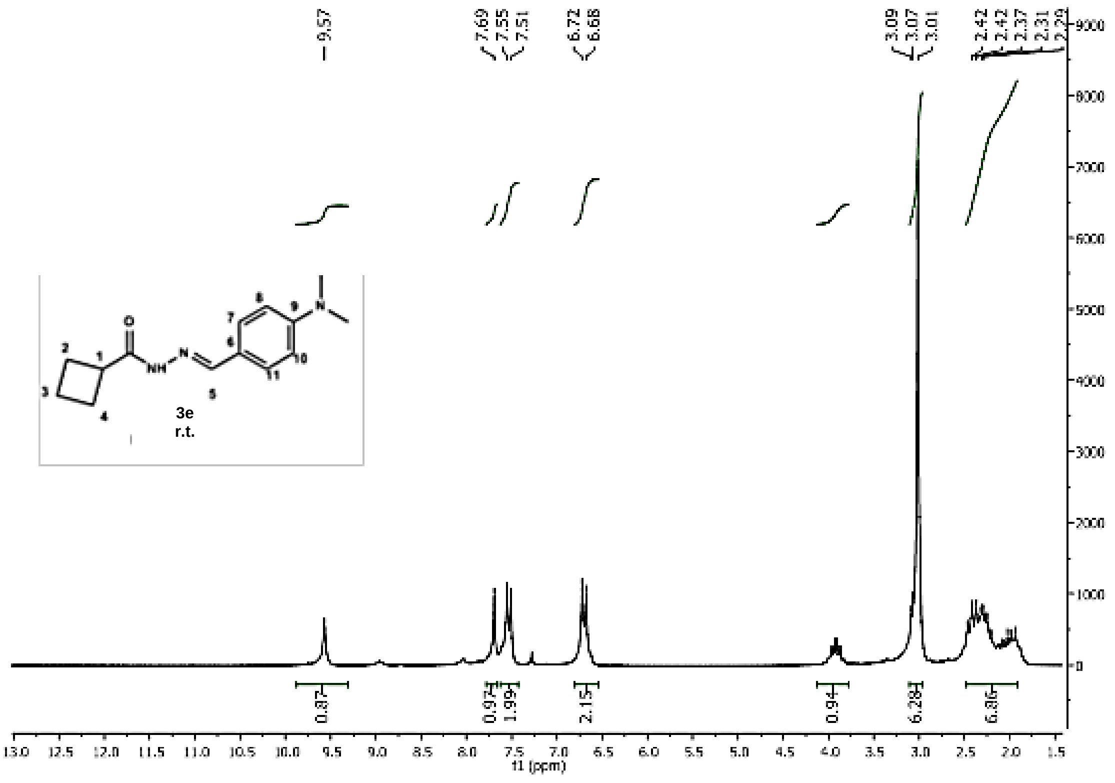

Figure S19. ^{1}H NMR spectrum of **3e** ($CDCl_3$, 200 MHz).

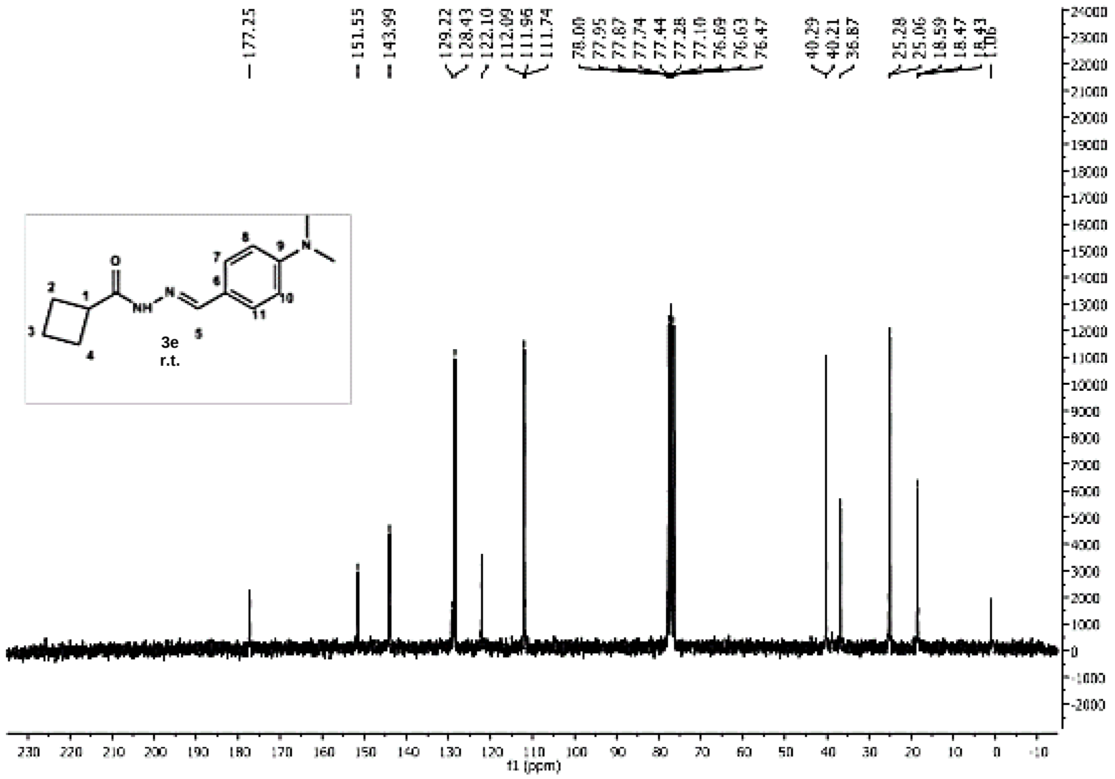

Figure S20. ^{13}C NMR spectrum of **3e** ($CDCl_3$, 50 MHz).

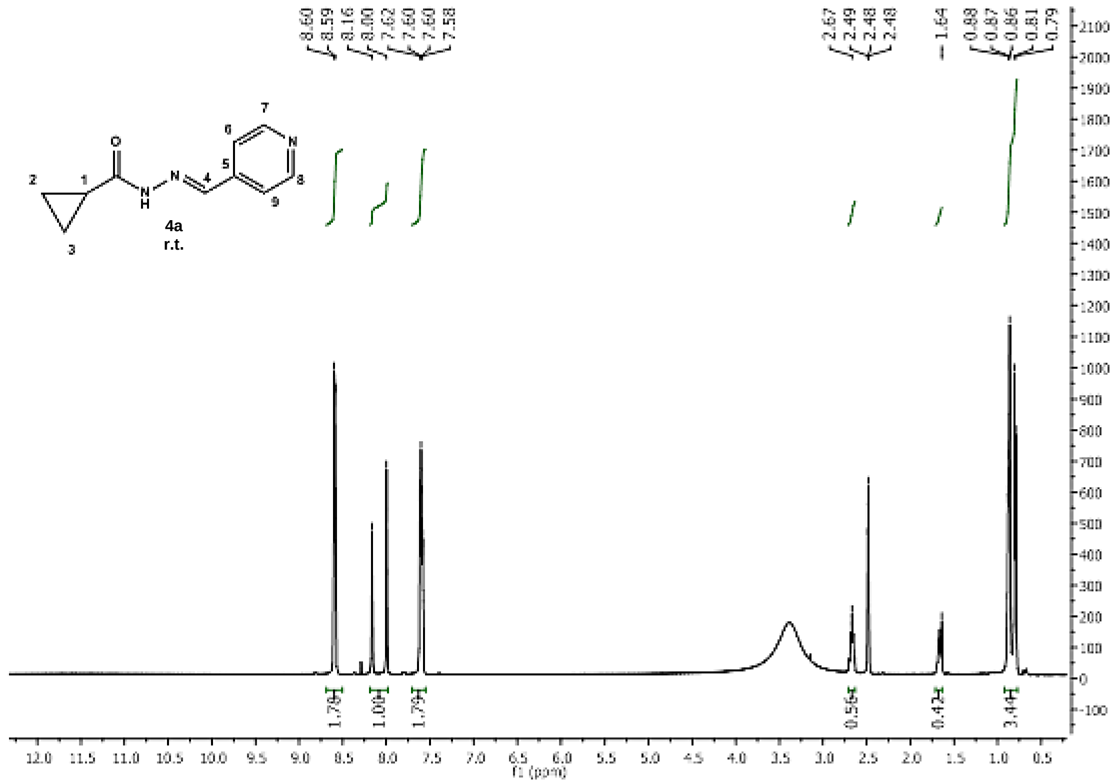

Figure S21. ^{1}H NMR spectrum of **4a** (DMSO-d_6, 200 MHz).

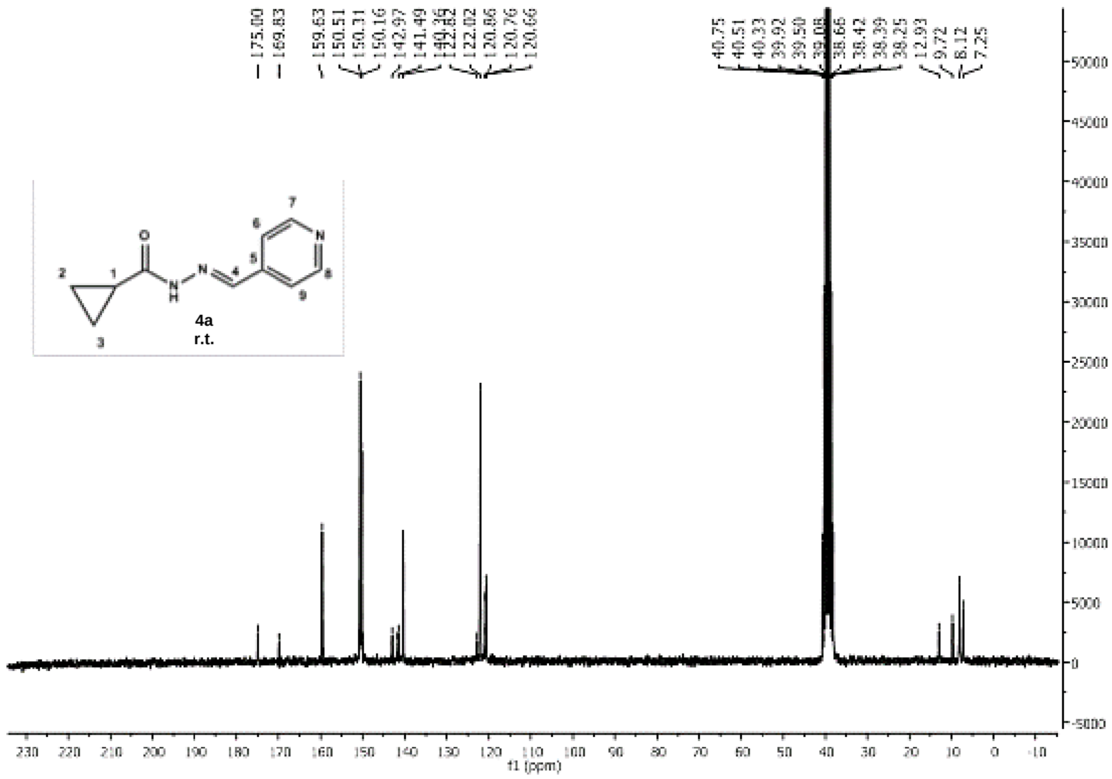

Figure S22. ^{13}C NMR spectrum of **4a** (DMSO-d_6, 50 MHz).

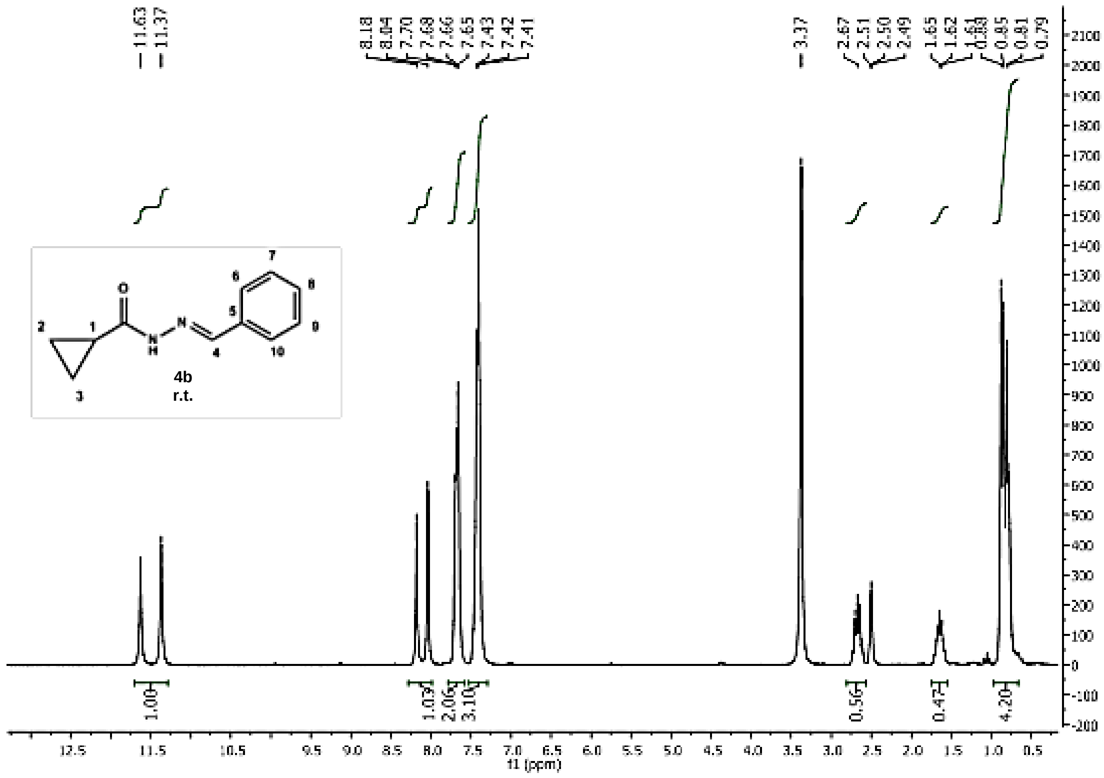

Figure S23. ^{1}H NMR spectrum of **4b** (DMSO-d_6, 200 MHz).

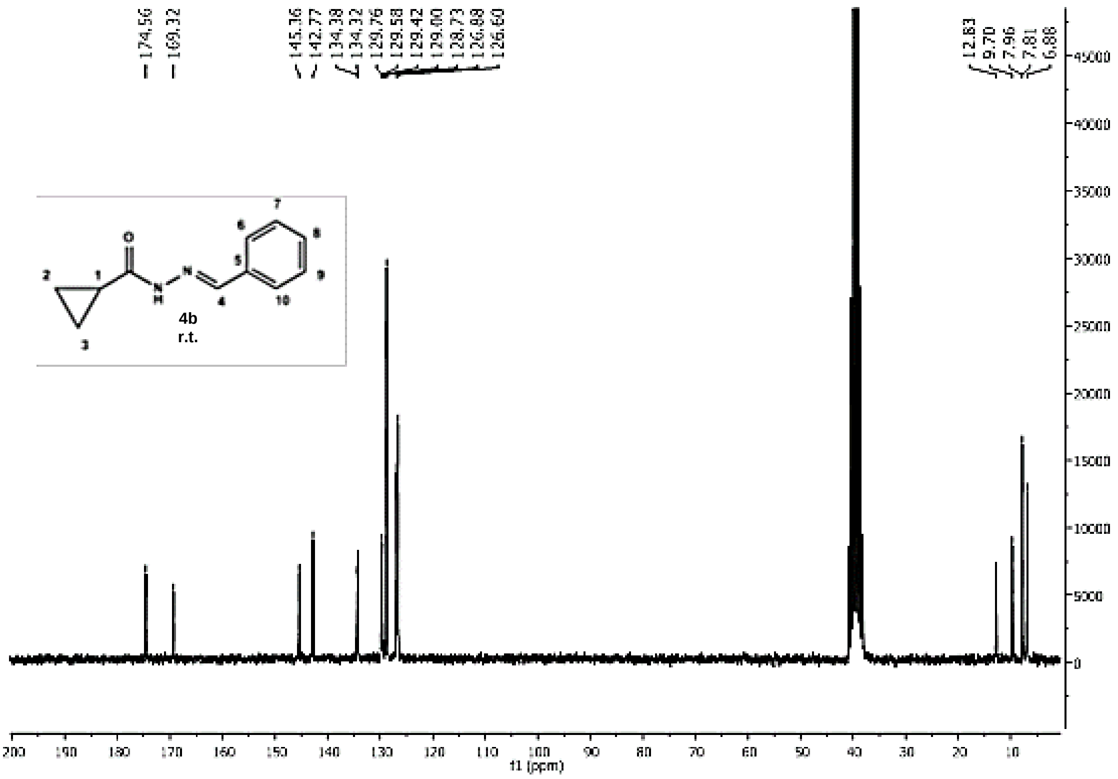

Figure S24. ^{13}C NMR spectrum of **4b** (DMSO-d$_6$, 50 MHz).

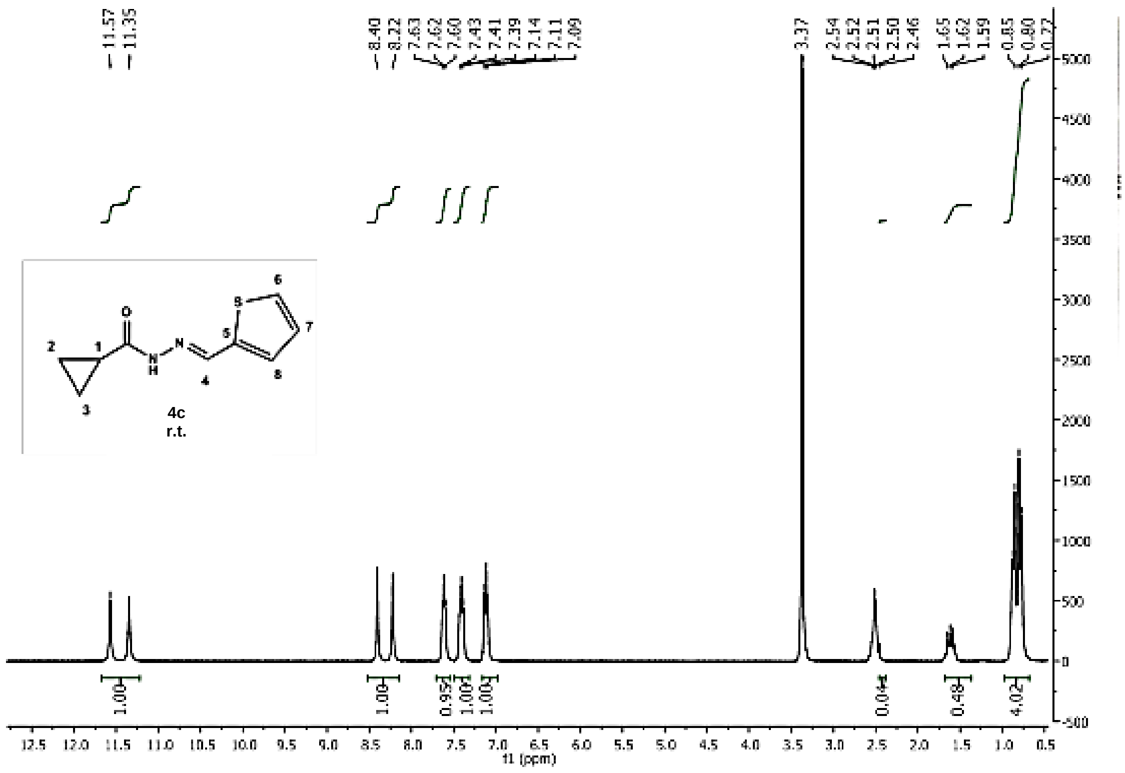

Figure S25. ^{1}H NMR spectrum of **4c** (DMSO-d$_6$, 200 MHz).

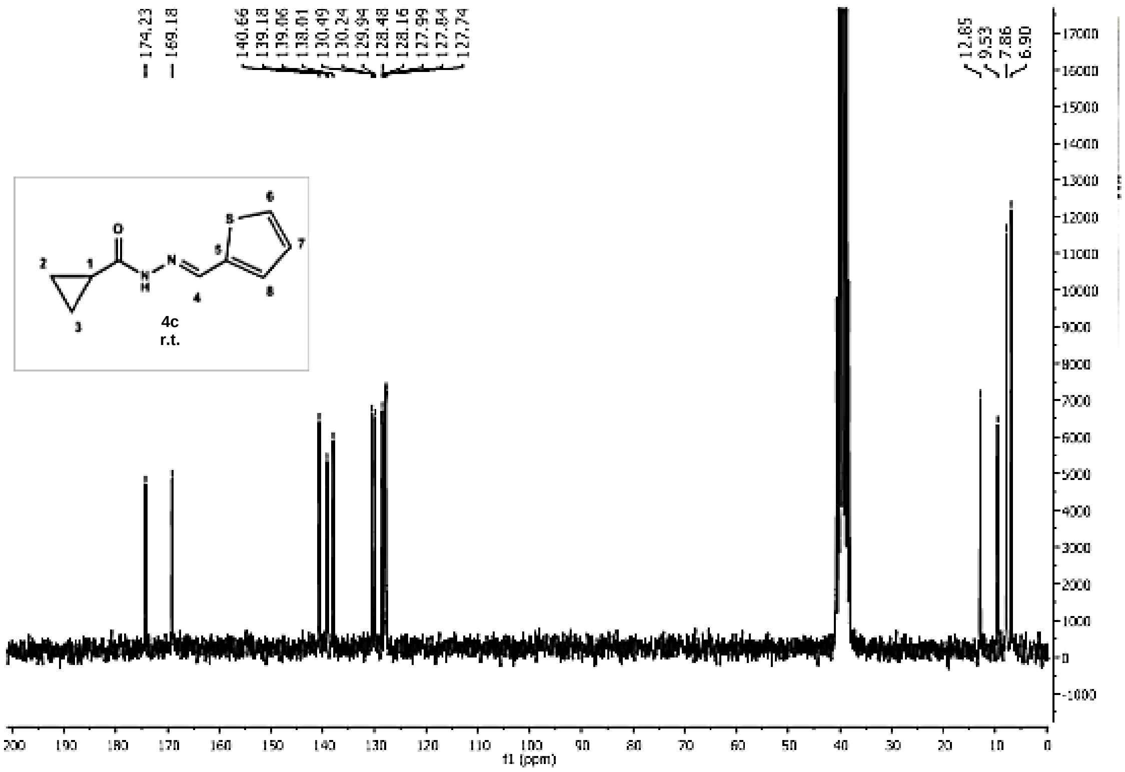

Figure S26. ^{13}C NMR spectrum of **4c** (DMSO-d_6, 50 MHz).

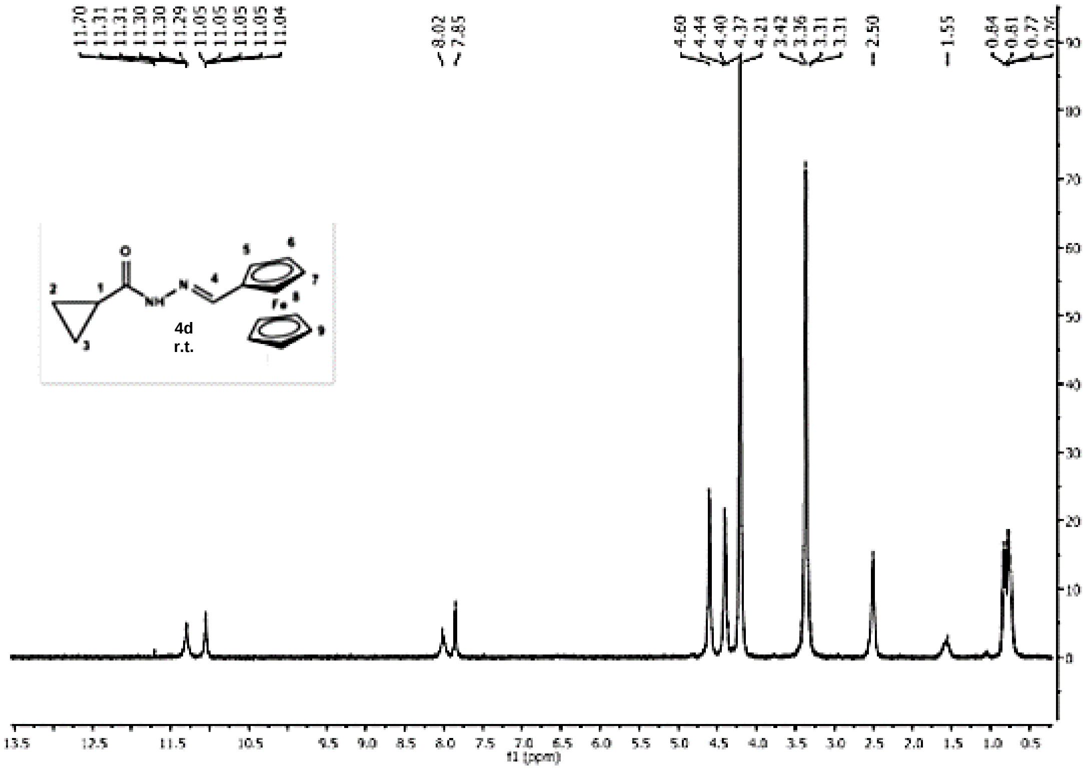

Figure S27. ^{1}H NMR spectrum of **4d** (DMSO-d_6, 200 MHz).

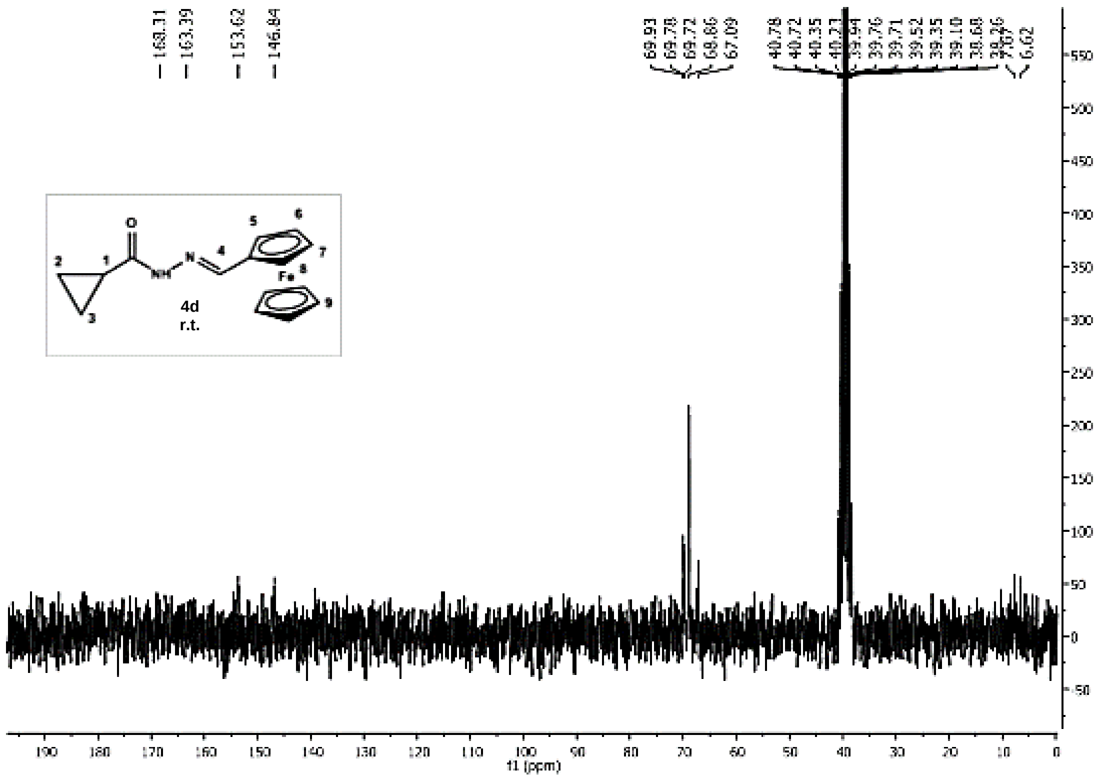

Figure S28. ^{13}C NMR spectrum of **4d** (DMSO-d$_6$, 50 MHz).

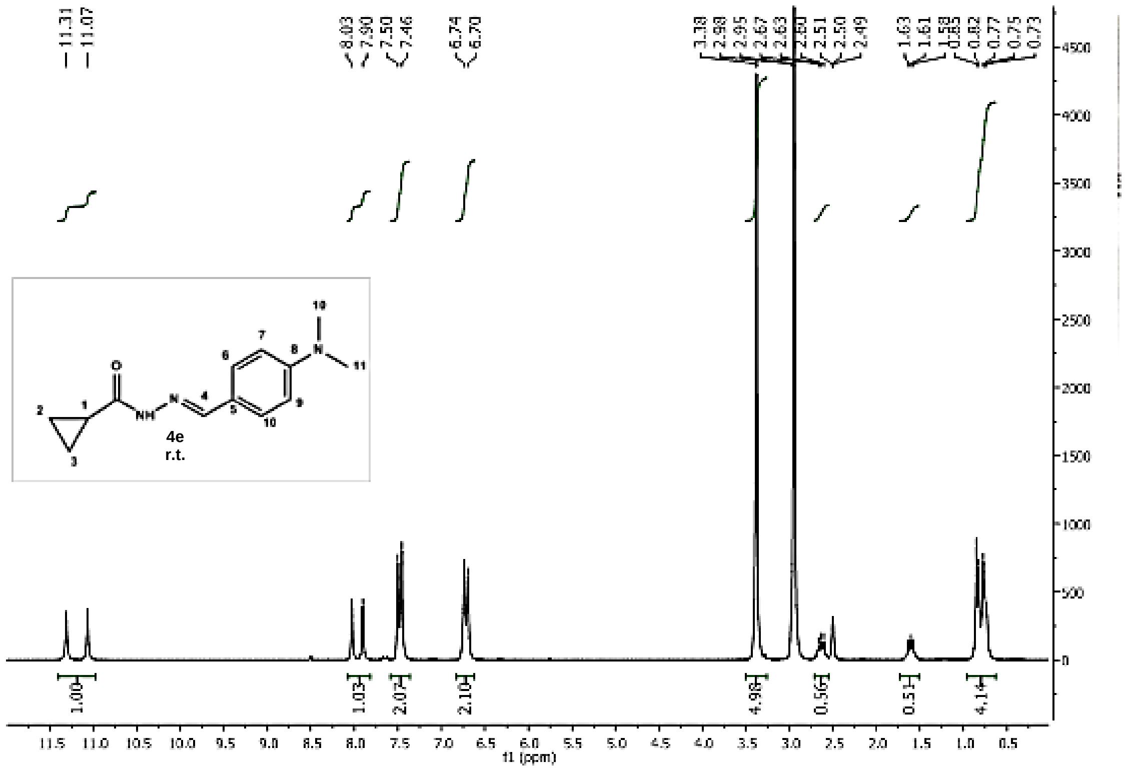

Figure S29. ^{1}H NMR spectrum of **4e** (DMSO-d$_6$, 200 MHz).

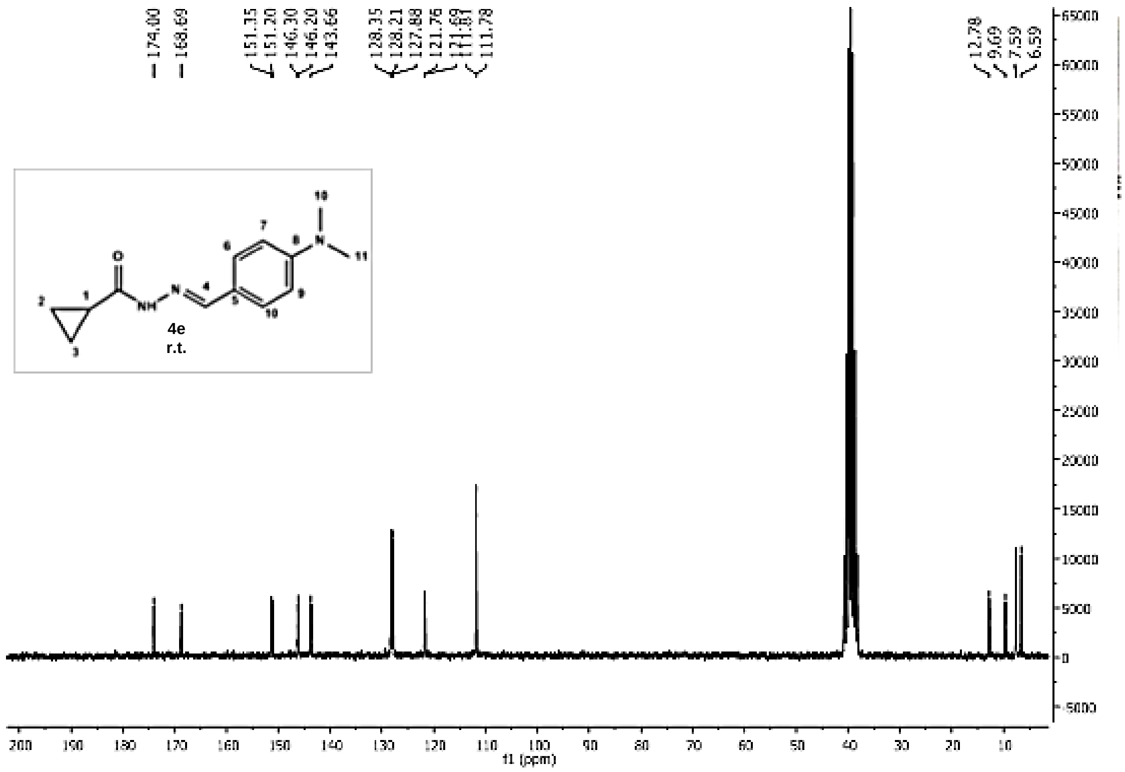

Figure S30. ^{13}C NMR spectrum of **4e** (DMSO-d$_6$, 50 MHz).

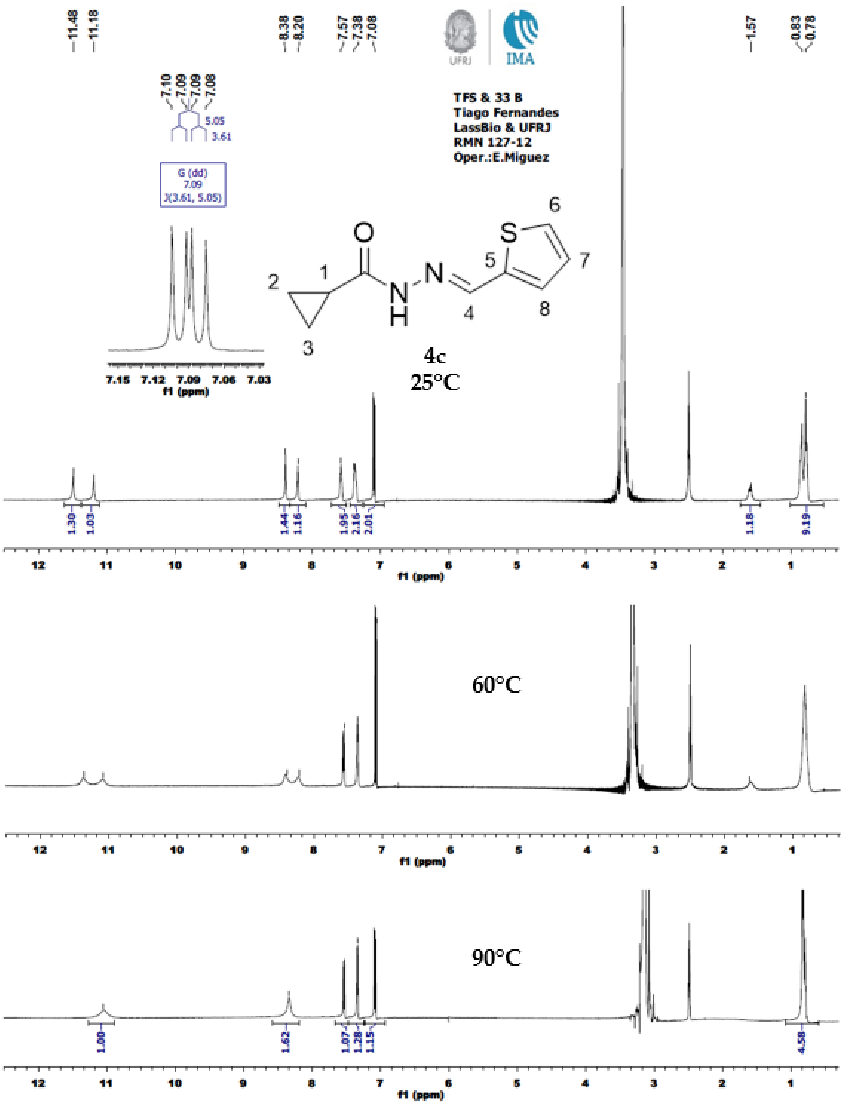

Figure S31. ^{1}H NMR spectra of **4c** at different temperatures (DMSO-d$_6$, 300 MHz).
